# Constraints to universal coverage: inequities in health service use and expenditures for different health conditions and providers

**DOI:** 10.1186/1475-9276-10-50

**Published:** 2011-11-13

**Authors:** Obinna Onwujekwe, Chima Onoka, Benjamin Uzochukwu, Kara Hanson

**Affiliations:** 1 Health Policy Research Group, Dept of Pharmacology and Therapeutics, University of Nigeria Enugu-Campus, Enugu, Nigeria; 2 Department of Health Administration and Management, University of Nigeria Enugu-Campus, Enugu; 3 Department of Community Medicine, College of Medicine, University of Nigeria Enugu-Campus, Enugu; 4 Department of Global Health and Development, London School of Hygiene and Tropical Medicine, London

**Keywords:** Health seeking, expenditures, inequities, economic burden

## Abstract

**Background:**

There is need for new information about the socio-economic and geographic differences in health seeking and expenditures on many health conditions, so to help to design interventions that will reduce inequity in utilisation of healthcare services and ensure universal coverage.

**Objectives:**

The paper contributes additional knowledge about health seeking and economic burden of different health conditions. It also shows the level of healthcare payments in public and private sector and their distribution across socioeconomic and geographic population groups.

**Methods:**

A questionnaire was used to collect data from randomly selected householders from 4,873 households (2,483 urban and 2,390 rural) in southeast Nigeria. Data was collected on: health problems that people had and sought care for; type of care sought, outpatient department (OPD) visits and inpatient department (IPD) stays; providers visited; expenditures; and preferences for improving access to care. Data was disaggregated by socio-economic status (SES) and geographic location (urban versus rural) of the households.

**Results:**

Malaria and hypertension were the major communicable and non-communicable diseases respectively that required OPD and IPD. Patent medicine dealers (PMDs) were the most commonly used providers (41.1%), followed by private hospitals (19.7%) and pharmacies (16.4%). The rural dwellers and poorer SES groups mostly used low-level and informal providers. The average monthly treatment expenditure in urban area was 2444 Naira (US$20.4) and 2267 Naira (US$18.9) in the rural area. Higher SES groups and urbanites incurred higher health expenditures. People that needed healthcare services did not seek care mostly because the health condition was not serious enough or they could not afford the cost of services.

**Conclusion:**

There were inequities in use of the different providers, and also in expenditures on treatment. Reforms should aim to decrease barriers to access to public and formal health services and also identify constraints which impede the equitable distribution and access of public health services for the general population especially for poor people and rural dwellers.

## Introduction

It is important to generate new information that will aid the better understanding of equity issues in health seeking patterns, expenditures, health financing and factors that can either enable or constrain the provision and utilisation of optimal quality health services for reducing the burden of communicable and non-communicable diseases. This is because, both communicable and non-communicable diseases constitute a considerable burden to households, especially in the present health system financing environment where households bear disproportionate proportion of health system costs arising from prevailing user fees regimen and dominance of out-of-pocket payments. Previous studies show that the burden from these diseases are considerable and increasing [1-4].

A constraining factor to access to health services is the negative effect of household health expenditures paid as user fees due to limited funding from government. Nonetheless, it has been shown that the higher the level of health expenditures by the government, the higher the disease burden declines [5]. Presently, public expenditure funded through general tax revenue in Nigeria accounts for only 20-30% of total health expenditure, while 70-80% comes from other sources, with the bulk of this taking the form of private payments [6,7]. Reliance on private payments transfers the financial burden on the poor [8,9].

Payments for health services, in the form of user charges present a barrier to access [10,11] and lead to increased disease burden. In most cases, user fees have occurred spontaneously as a result of: the scarcity of public financing; the prominence of the public system in the supply of essential healthcare; the government's inability to allocate adequate financing to its health system; the readiness of the poor and the better-off to pay fees as a way of reducing the travel and time costs of alternative sources of care; the low salaries of health workers; the limited public control over pricing practices by public providers; and the lack of key medical supplies such as drugs [12].

Previously, available data in Nigeria show that user fees paid mostly through out-of-pocket spending (OOPS) is an impediment to access to services [13,14]. However, there are other factors related to poverty, geographic inaccessibility and lack of knowledge [11,15]. The 2008 NDHS in Nigeria showed the most important barrier that women faced in accessing healthcare was getting money for treatment [4]. The other reasons that the women mentioned included lack of drugs in health facilities, transportation costs, distance to health facility and not having a provider available in the health facilities [4].

In efforts to improve health care financing, provision and utilisation of health services in the country, some interventions have been started. For instance, the formal sector programme of the National Health Insurance Scheme (NHIS) and community-based health insurance (CBHI) schemes are important means of improving access to health services [16]. The coverage of the NHIS programme is quite low (less than 5% of the population) and the CBHI schemes only exist in a pocket of communities covering less than 1% of the population [17]. Hence, these schemes have not started having significant effects on improving health seeking in the country.

The paper contributes additional knowledge about health seeking and economic burden of different health conditions. It also shows the level of healthcare payments and their distribution across socioeconomic and geographic population groups. There is great need for such new information about the socio-economic and geographic differences in household health seeking, expenditures on many diseases and health conditions, to aid the design of interventions that will ensure universal coverage with healthcare services.

## Research methods

### Research Area

The research was undertaken in 4 selected Local Government Areas (LGAs); 2 rural and 2 urban LGAs from Enugu and Anambra states (2 LGAs per state). The two state capitals were selected as the urban LGAs and two rural LGAs were purposively selected. Enugu is the capital city of Enugu state. There are 17 LGAs in the state, of which 5 are urban. Enugu state has an estimated population of about 3,100,000 (projected from 1991 census). Anambra state has a population of 4,054,824 (projected from 1991 census). Its capital city is Awka, and it is comprised of 21 LGAs, 6 of which are urban. Each state capital has a tertiary hospital and each urban LGA has a public hospital. There are health centres in all rural LGAs. The private sector is represented by private hospitals, clinics, pharmacies, Patent Medicine Dealers (PMDs), and mission hospitals, all of which are found in both states.

### Data Collection

A pre-tested questionnaire was administered by trained field workers to a minimum sample of 4,800 randomly selected householders from 4 LGAs (1,200 people per LGA). The sample size per state was determined using: the estimated number of households in the urban and rural LGAs per state - which is approximately 1 million (with an average household size of 5 people); a power of 80% and 95% confidence level. In each selected household, one woman (the primary care giver) - or in her absence - the male head of the household was interviewed. This study was carried out between 2008 and 2009.

Data was collected on: health problems that people had and sought care for; type of care sought in terms of outpatient department (OPD) visits and inpatient department (IPD) stays; types of providers visited; level of expenditures; and preferences for improving access to care. The average expenditure by households on OPD and IPD were elicited. Trends in health seeking for both OPD visits and IPD stays were elicited. Data was also collected on household ownership of assets and food expenditure. A one-month recall period was used to collect information on household healthcare expenditure on OPD, as well as mode of payment for health expenditure. The one-month recall period reduced the incidence of recall bias that would occur if longer periods were used. However, a six-month recall period was used for collecting data on IPD stays because such events are rarer than out-patient visits.

### Data analysis

The levels of occurrence of various health conditions/diseases were calculated, and the types of services that were accessed and providers that people visited were analysed. In addition, the level of healthcare expenditure on various health conditions/diseases and providers were estimated. The specific indicators were the level of health service use per month for household members (number of OPD visits and IPD admissions) aggregated to the household level, and total monthly health expenditure per household.

The mean monthly expenditure per household was compared between different SES groups and between urban and rural dwellers. The specific indicators were: level of household expenditure on OPD and IPD; SES and rural-urban differences in the use of different health financing mechanisms; average monthly household expenditure across different healthcare providers by different financing mechanisms; and average household expenditure on different diseases and health conditions.

Note: 1US$ = 120 Naira

Principal components analysis (PCA) was conducted using STATA [18] to create a SES index [19] (Filmer and Pritchett, 2001) using information on the households' ownership of a radio, bicycle, motorcycle, car, refrigerator, generator, kerosene lamp, together with the weekly household cost of food. The index generated was used to divide the households into five equal-sized SES groups (quintiles). The quintiles were Q1 (most poor), Q2 (very poor), Q3 (poor), Q4 (less poor), and Q5 (least poor). The measure of inequity in household healthcare payments was the concentration index [20,21]. The concentration index varies from -1 and +1. A negative sign denotes that the distribution of the variable of interest favours the poor, and if positive, it means that it favours the least poor [22].

Frequency distributions of the variables by SES and rural-urban location were calculated and chi-squared (*X*^2^) tests of trend analysis for statistical difference were undertaken. The Kruskal-Wallis non-parametric test, which reports a *X*^2 ^statistic, was used to compare differences in means of continuous variables. The Kruskal-Wallis is the non-parametric equivalent of ANOVA.

## Results

### Socio-economic and demographic characteristics

There were 2,390 rural households and 2,483 urban households. The average household size was 4.5 people (Table 1). The mean age of the respondents was 41.6 years. Majority of the respondents were female and had some formal education. Household weekly food expenditure was 3,143 Naira from the combined data, but it was higher in the urban areas compared to the rural areas. Annual household non-food expenditure was 95,029 Naira, but again it was higher in the urban areas compared to the rural areas. Most of the households owned functional radios and kerosene lamps. Bicycles, motorcycles, cars, and generators were the least commonly owned household assets.

**Table 1:** Respondents' and households' socio-economic and demographic characteristics

	Urban N = 2,483 (51%)	Rural N = 2,390 (49%)	Combined N = 4,873
No. of household residents: Mean (SD)	5.1(2.0)	3.9 (1.9)	4.5 (2.0)

Age of respondent: Mean (SD)	37.3 (14.0)	46.0 (15.6)	41.6 (15.4)

Sex (Female): n (%)	2,180 (87.8)	2,203 (88.7)	4,383 (89.9)

Attended school: n (%)	2,250 (90.6)	1,336 (53.8)	3,586 (73.6)

Years of education: Mean (SD)	12.0 (3.9)	8.6 (3.6)	10.9 (4.1)

Average weekly food expenditure: mean (SD)	3,760.9 (3,801.5)	2,502.5 (2,702.9)	3,143.2 (3,367.2)

Average weekly food cost: mean (SD)	3,817.7 (3,831.5)	3,154.9 (3,000.3)	3,492.6 (3,464.5)

Per capita weekly food expenditure: Mean (SD)	827.8 (904.6)	734.4 (1,056.1)	782.0 (982.9)

Per capita weekly food cost: Mean (SD)	841.4 (913.4)	919.5 (1,132.5)	879.7 (1,027.3)

Household owns a radio: n (%)	2,210 (89.0)	1,975 (79.5)	4,185 (86%)

Household owns a fridge: n (%)	1,792 (72.2)	426 (17.2)	2,218 (45.6)

Household owns a TV: n (%)	2,234 (90.0)	1,145 (46.1)	3,379 (69.5)

Household owns a bicycle: n (%)	78 (3.1)	598 (24.1)	676 (13.9)

Household owns a motorcycle: n (%)	298 (12.0)	419 (16.9)	717 (14.7)

Household owns a car: n (%)	679 (27.3)	131 (5.3)	810 (16.7)

Household owns a kerosene lamp: n (%)	2,402 (96.7)	2,344 (94.4)	4,746 (97.6)

Household owns a generator: n (%)	519 (20.9)	323 (13.0)	842 (17.3)

Household owns a rechargeable lamp: n (%)	963 (38.8)	465 (18.7)	1,428 (29.5)

SES quintiles (asset index)			

Q1 (most poor)	127 (5.1)	848 (34.2)	975 (20)

Q2 (very poor)	310 (12.5)	664 (26.7)	974 (20)

Q3 (poor)	542 (21.8)	433 (17.4)	975 (20)

Q4 (less poor)	754 (30.4)	221(8.9)	975 (20)

Q5 (least poor)	750 (30.2)	224 (9.0)	974 (20)

### General health services use

Among the surveyed population there were 5,292 OPD visits and 282 IPD stays in the month preceding the survey. Some were multiple visits but 3936 respondents made at least one visit to the different providers. Malaria was the major health condition that required both OPD and IPD visits by households (Table 2). The next most common health condition was respiratory diseases. Hypertension was the number one non-communicable disease cause of both OPD and IPD visits. Only one household reported a visit due to HIV/AIDS.

**Table 2:** Occurrence of different diseases states/health conditions in households that required outpatient visits and in-patient admissions

	Outpatient visits n (%)	Inpatient admissions n (%)
Malaria	2,694 (51.4)	93 (33)

Respiratory diseases	937 (17.7)	26 (9.2)

Diarrhoea	296 (5.6)	21 (7.4)

Diabetes	73 (1.4)	4 (1.4)

Cancer	4 (0.1)	2 (0.7)

Hypertension	140 (2.7)	14 (5)

Trauma	86 (1.6)	13 (4.6)

Immunisation	90 (1.7)	1 (0.4)

HIV	1 (0.02)	0 (0)

Appendicitis	13 (0.25)	17 (6.0)

ANC	74 (1.4)	7 (2.5)

Childbirth	27 (0.5)	22 (7.8)

Others	1,701 (32.1)	62 (22.0)

The private sector was by far the most common source of healthcare. PMDs were the most common providers visited for healthcare followed by private hospitals and pharmacies (also in the private sector) (Table 3). The public hospitals and PHC centres were used to a lesser degree by the households.

**Table 3:** Providers visited for healthcare services

	n (%)
Patent medicine dealer	1,613 (41.1)

Private hospital	735 (19.7)

Pharmacy	645 (16.4)

Public hospital	547 (13.9)

Primary healthcare centre	126 (3.2)

Herbalist	105 (2.7)

Home	23 (1.6)

Laboratory	12 (0.3)

Others	120 (3.1)

The urbanites made greater use of private and public hospitals, pharmacies, and herbalists than rural dwellers (p < 0.05). Conversely, the rural dwellers were more likely to use PMDs. The use of public and private hospitals as well as pharmacies increased with increased SES, whilst the use of PMDs decreased with increasing SES (p < 0.05). These socio-economic differences are confirmed by the concentration indices, which are negative (i.e. pro-poor) for PHC centres, PMDs, laboratories, and others; and positive (pro-rich) for home care, private hospitals, public hospitals, pharmacies and herbalists (Table 4).

**Table 4:** Differential use of different providers by different population groups

	Home n(%)	Private hospital n(%)	Public hospital n(%)	PHC n(%)	PMD n(%)	Pharm-acy shop n(%)	Herbalist n(%)	Lab n(%)	Others n(%)
Differential use of different providers by urban-rural residence									

Urban	15 (65%)	479(65%)	390 (71%)	55 (44%)	760 (47%)	579 (90%)	89 (85%)	5 (42%)	49 (41%)

Rural	8 (35%)	256 (35%)	157 (29%)	71 (56%)	853 (53%)	66 (10%)	16 (15%)	7 (58%)	71 (59%)

X2 (p-value)	1.4 (.29)	68.6 (.0001)	100.6 (.0001)	2.9 (.10)	15.3 (.0001)	446.0 (.0001)	48.6 (.0001)	.43 (.57)	5.2 (.026)

Differential use of different providers by SES									

Quintile 1	4 (17%)	94(13%)	44(8%)	28(22%)	376(23%)	42(7%)	9(8.5%)	2 (17%)	32 (26.5%)

Quintile 2	4(17%)	108(15%)	77(14%)	28(22%)	376(23%)	66(10%)	9 (8.5%)	5 (42%)	36 (30%)

Quintile 3	3(14%)	149(20%)	124(23%)	26(21%)	315(20%)	157 (24%)	29 (28%)	2 (17%)	23 (19%)

Quintile 4	6(26%)	171(23%)	175(32%)	27(21%)	302(19%)	181 (28%)	18 (17%)	1 (8%)	20 (17%)

Quintile 5	6(26%)	213(29%)	127(32%)	17(14%)	244(15%)	191 (30%)	40 (38%)	2 (17%)	9 (8.5%)

CI	0.09	0.18	0.19	-0.07	-0.09	0.25	0.25	-0.13	-0.22

X2 (p-value)	1.2 (.88)	74.5 (.00001)	105.6 (.00001)	3.5 (.48)	55.4 (.0001)	180.3 (.00001)	35.4 (.0001)	3.8 (.43)	19.0 (.001)

Total	23	735	547	126	1613	645	105	12	120

### Expenditures on healthcare seeking

The mean monthly household health expenditure was 2,354 Naira (SD 6,080 Naira). Of this the mean monthly household health expenditure in public health facilities was 661 Naira (SD 3,446 Naira). The remaining expenditure was incurred in the private sector. The average monthly household expenditure on outpatient care was 1,809 Naira, and about 610 Naira for inpatient care (Table 5). Average monthly household expenditure was highest in public hospitals (423 Naira for outpatient care, and 230 Naira for inpatient care), compared with PHC (48 Naira for outpatient care and 5 Naira for inpatient care).

**Table 5:** Average monthly total household expenditures on OPD and IPD in public health facilities

	Mean (SD) Naira	US$
Total health expenditures	2353.8 (6079.7)	19.6

Expenditure in public facilities	661.3 (3,445.7)	5.5

Total expenditure - outpatient care	1809.0 (4,612.0)	15.1

Total expenditure - inpatient care	609.6 (4,249.1)	5.1

Expenditure on outpatient care in public facilities	457.8 (2,115.5)	3.8

Expenditure on inpatient care in public facilities	203.5 (2,725.9)	1.7

Expenditure on outpatient care in public hospital	422.7 (2,022.2)	3.5

Expenditure on outpatient care in PHC	48.3 (724.7)	0.4

Expenditure on inpatient care in public hospital	229.6 (3,233.0)	1.9

Expenditure on inpatient care in PHC	4.6 (144.1)	0.04

Table 6 shows that average monthly household outpatient care expenditure for those with chronic diseases such as diabetes and hypertension was more than 4000 Naira. However, in general, inpatient care expenditures were higher than outpatient care expenditures, but the frequency of use of outpatient care was much more than inpatient care.

**Table 6:** Mean monthly household expenditures for different diseases states/health conditions

	Outpatient care Mean (SD)	Inpatient care Mean (SD)	Transportation Mean (SD)
Malaria	1407.0 (2,594.5)	12,442.0 (16,263.3)	198.3 (680.9)

Respiratory diseases	1,241.1 (3,359.5)	10,023.1 (8,702.7)	198.6 (323.1)

Diarrhoea	1,395.9 (2,598.7)	7,995.7 (4,429.2)	180.1 (212.3)

Diabetes	4,957.8 (5,820.8)	21,900.0 (14,396.8)	785.5 (1,574.2)

Cancer	1,725.0 (1,330.7)	17,900.0 (13,010.8)	433.3 (208.2)

Hypertension	5,843.1 (7,362.7)	13,575.0 (13,575.1)	468.9 (1,059.2)

Trauma	4,357.0 (11,948.8)	21,462.3 (22,877.6)	617.7 (880.4)

Immunisation	463.2 (692.2)	0	132.2 (151.3)

HIV	0	0	0

Appendix	6,926.9 (7,141.3)	18,185.3 (26,276.4)	625.0 (682.3)

ANC	1,524.2 (1,677.6)	16,021.4 (16,483.3)	215.9 (314.3)

Childbirth	4,226.5 (5,721.5)	20,183.9 (28,057.4)	360.8 (330.8)

Others	2,193.2 (5,138.4)	24,817.5 (42,602.9)	326.5 (928.1)

Table 7 shows that the urban dwellers had higher average monthly household expenditure compared to the rural dwellers. The differences in monthly household expenditures in the public sector for both OPD visits and IPD stays were not statistically different between the urban and rural areas. Table 7 also shows that the higher the SES, the higher the total health expenditures, expenditures in the public sector, and expenditures on OPD visits in the public sector. The monthly expenditure on IPD was not statistically different across the quintiles.

**Table 7:** Monthly household health expenditures in public facilities for the whole sample

	Total expenditure in public and private facilities Mean (SD)	Total expenditure in public facilities Mean (SD)	OPD Public Mean (SD)	IPD Public Mean (SD)
by urban-rural				

Urban	2,443.8 (6,166.6)	620.0 (3,199.5)	439.0 (2,160.8)	180.9 (2,347.8)

Rural	2,266.5 (5,993.7)	700.8 (3,666.6)	475.7 (2,070.9)	225.2 (3,045.5)

X2 (p-value)	16.8 (.0001)	1.5 (.22)	1.4 (.24)	.06 (.81)

by SES				

Quintile 1	1,868.3 (5,184.7)	392.2 (1,901.0)	338.6 (1,678.8)	53.6 (876.6)

Quintile 2	2,256.1 (5,984.3)	702.2 (4,189.7)	450.5 (2,462.2)	251.6 (3,423.1)

Quintile 3	2,396.8 (6,178.3)	699.8 (3,623.1)	439.1 (1,818.5)	260.7 (3,095.1)

Quintile 4	2,260.7 (5,713.4)	702.4 (2,857.3)	577.5 (2,199.4)	125.0 (1,851.4)

Quintile 5	2,987.4 (7,128.2)	810.1 (4,106.3)	483.4 (2,311.7)	326.7 (3,412.1)

X2 (p-value)	9.77 (.045)	16.0 (.003)	15.8 (.003)	6.5 (.17)

CI	0.08	0.10	0.07	0.17

Table 8 recalculates mean expenditure for those households that incurred positive expenditure. Urban dwellers had higher expenditure for both outpatient and inpatient care compared to the rural dwellers, but the p-value was not significant for inpatient care. The table also shows that the higher the SES, the higher the amount of money spent on outpatient care. This pattern was also seen for inpatient care, except that expenditure was highest in quintile 3. Overall, the concentration index for expenditure is positive for both inpatient and outpatient expenditure.

**Table 8:** Monthly health expenditure in public facilities fore households which incurred expenditure > 0.

	Outpatient care, public sector facilities Mean (SD)	Inpatient care, public sector facilities Mean (SD)
**By urban-rural areas**		

Urban	2,859.0 (66,829.0) n = 3,074	19,603.1 (31,354.6) n = 94

Rural	1,622.1 (4,089.3) n = 2,322	15,153.0 (24,241.5) n = 127

X2 (p-value)	22.1 (.00001)	2.1 (.15)

**by SES**		

Quintile 1	1,393.9 (3,972.7) n = 1,183	14,004.1 (19,706.5) n = 39

Quintile 2	1,511.2 (4,249.0) n = 1,061	10,860.0 (11,769.1) n = 40

Quintile 3	1,644.3 (3,664.2) n = 1,042	22,280.6 (39,679.3) n = 40

Quintile 4	1,741.5 (3,711.1) n = 1,129	18,240.5 (30,125.0) n = 51

Quintile 5	1,973.0 (4,517.6) n = 970	19,140.6 (27,197.0) n = 50

X2 (p-value)	37.8 (.00001)	7.7 (.10)

CI	0.07	0.08

Table 9 shows how spending on the different providers differs by population group. Urban dwellers spent more money than rural dwellers on public and private hospitals, pharmacies, and laboratories. The table also shows that as SES increases, the expenditures on public and private hospitals, pharmacies, laboratories, and the home increases. Conversely, as SES decreases, expenditures on PHC centres, PMDs and herbalists increases.

**Table 9:** Mean monthly total household expenditure on treatment paid to different providers

	Home	Private hospital	Public hospital	PHC centres	PMD	Pharmacy	Herba-list	**Lab**.	Others
Mean expenditures on treatment by urban-rural residence									

Urban	18.7 (467.1)	1,320.8 (6,357.4)	720.8 (3,595.1)	24.5 (2,98.1)	231.9 (934.5)	283.1 (1,031.8)	2.0 (48.3)	60.4 (442.9)	207.9 (5,424.1)

Rural	1.5 (44.6)	624.3 (3,776.2)	427.1 (4,970.4)	46.1 (450.4)	300.2 (1,949.0)	78.1 (1,193.9)	3.2 (112.6)	23.6 (441.8)	260.8 (3,233.9)

X2 (p-value)	1.4 (.23)	68.1 (.0001)	98.4 (.0001)	3.0 (.082)	12.7 (.0001)	435.4 (.0001)	0.43 (.51)	48.4 (.0001)	5.3 (.022)

Mean expenditures on treatment by SES									

Quintile 1	2.3 (64.5)	380.5 (1,766.5)	161.9 (1,194.4)	53.8 (582.7)	340.6 (2,668.9)	99.0 (1,142.3)	5.6 (172.1)	44.0 (661.5)	263.2 (3,641.2)

Quintile 2	4.4 (120.8)	721.7 (4,411.1)	417.8 (3,338.5)	54.5 (468.1)	285.0 (1,020.8)	120.7 (1,376.8)	2.4 (42.7)	19.7 (297.9)	281.0 (2,718.9)

Quintile 3	1.0 (25.0)	903.2 (3,797.3)	608.3 (3,731.4)	19.4 (186.9)	269.2 (1,359.7)	180.9 (700.1)	1.1 (24.6)	45.9 (375.7)	481.4 (8,821.6)

Quintile 4	32.1 (698.9)	1,172.1 (4,748.1)	926.2 (6,521.5)	28.0 (270.7)	264.7 (1,076.7)	243.0 (956.1)	1.5 (45.0)	31.1 (259.7)	82.9 (1,196.6)

Quintile 5	11.5 (232.4)	1,726.4 (8,854.9)	774.2 (4,929.5)	19.4 (233.4)	166.4 (576.7)	271.5 (1,277.9)	2.4 (53.9)	71.6 (494.3)	59.3 (893.3)

X2 (p-value)	1.2 (.88)	75.8 (.0001)	105.3 (.0001)	3.6 (.47)	52.9 (.0001)	177.0 (.0001)	3.8 (.44)	35.2 (.0001)	19.0 (.0001)

CI	0.36	0.26	0.24	-0.22	-0.11	0.20	-0.25	0.13	-0.21

**Total**	10.3 (335.1)	979.8 (5,265.7)	577.2 (4,324.6)	35.1 (380.4)	265.3 (1,518.2)	182.8 (118.6)	2.6 (86.0)	42.4 (442.7)	233.8 (4,488.6)

### Reasons healthcare was not sought and preferences for improving financing, provision and utilisation of healthcare services

The major reasons that people who needed healthcare services did not seek care were that either the condition was not serious enough (67.3%) or they could not afford the cost of services (35.5%). Other reasons given were that individual could not afford transport costs (9.1%); poor quality of services (6.8%); problems with geographic accessibility (4.6%); and others.

The three main suggestions that respondents gave for improving provision, utilisation and financing of healthcare services were provision of free services (78.6%), subsidising healthcare (55.3%), and construction of more public hospitals (50.7%). Other reasons given were improvement of quality of services in existing facilities (44.4%), provision of more health centres (40.8%), use of health insurance (14.3%), construction of more private hospitals (12.5%) and others (2.0%).

## Discussion

The findings show that there were significant inequities in use of the different providers, and in expenditures on treatment. Health seeking for fever or presumptive malaria was the most common motive for both OPD visits and IPD stays. Hypertension was the most common non-communicable diseases that required OPD visits and IPD stays. The fact that malaria was the most common public health problem and disease burden has been found in several other studies in Nigeria [1,3,4,23]. This reinforces the importance of tackling malaria due to its potential deplete household resources. However, it is surprising that despite the enormous amounts of money and other resources that have been invested in malaria control in Nigeria, the disease still remains the major reason for both outpatient visits and hospital stays.

There were striking inequities in use of the different providers, with the rural dwellers and poorer SES groups more likely to use low-level and informal providers, where treatment is usually of questionable quality [24,25], with the exception of herbalists that were used mostly by the middle and highest quintiles in this study. These low-level providers included the PMDs, herbalists, the health posts, and other drug sellers. Similar findings have been found in other studies in Nigeria and elsewhere [25-29]. Hence, despite all the interventions to expand the number and quality of public health facilities in the study area, their use was still lower than those of the private sector. Patent Medicine Dealers (PMDs) followed by private hospitals and pharmacy shops were the most commonly used healthcare providers. This has also been found in other studies in Nigeria and in other sub-Saharan African (SSA) countries [1-3,26]. The implication of this is that the poor and rural people access more inappropriate healthcare services, which predisposes them to spending more on services that are not beneficial - leading to economic loss and by extension a higher economic burden of illness.

Higher expenditures were incurred by urbanites and the better-off SES groups and the level of expenditure on healthcare services was generally quite high, for both OPD and IPD. Other studies showed that the better-off SES spend more on healthcare [26]. However, for all the health conditions, the average expenditures on IPD care were more than for OPD as expected, since inpatient care involves hospital stays and is usually for more serious conditions that require more and possibly more expensive drugs. However, because there were much more frequent outpatient visits compared to inpatient stays, the aggregate expenditure on outpatient care was higher. Another study in Nigeria found that the average monthly household healthcare expenditure in Enugu for 1^st ^and 5^th ^quintile were 53 Naira and 1,065 Naira respectively [30], and these were lower than the expenditures found in this study. The high level of expenditures probably deterred many households especially the most-poor and rural dwellers from accessing good quality providers. Some authors from their meta-analysis also found that user fee was a barrier to access to services [10].

It was not surprising to find that the lowest average expenditures were incurred in low level providers such as herbalists and PMDs and the highest average expenditure was incurred in private hospitals, followed by public hospitals. This disparity in expenditures could stem from the type of services offered by the different providers and particular treatment provision behaviours of the different providers. A study in Greece also shows significantly higher cost of using private facilities where services are perceived to be better [31]. Patients were found to make informal payments in order to receive better quality service [32]. The limited range of services offered by the low level providers, the low levels of their operating costs and their practices of providing incomplete services such as under-dosing with drugs and treatment based on clients' requests instead of using appropriate standard operating procedures could account for the lower level of expenditure at these providers [33].

As expected, the highest average expenditures were generally incurred for non-communicable diseases, although the expenditures for communicable diseases such as malaria were also quite high. However, it should be borne in mind that the vast majority of people sought treatment for and incurred expenditures on communicable diseases. Hence, the total expenditures on communicable diseases were more than that of non-communicable diseases. The higher average monthly household expenditures on non-communicable diseases is explained by the fact that most of them are chronic and require daily medication and regular visits to healthcare providers. The drugs that are required to treat or control them are also usually more expensive than drugs used for the treatment of communicable diseases, which are mostly acute in nature and are usually cured with one round of appropriate treatment.

The finding that expenditures OPD in public hospitals and total expenditure increased as SES quintile increased could be an income effect since the poorer quintiles are constrained by their budgetary limits to spend less on healthcare and also possibly travel shorter distances or use less comfortable but cheaper means of transportation to visit healthcare providers. The budget constraints on the poorer quintiles will most likely predispose them to accessing and consuming poor, incomplete and inappropriate treatment services, with possible dire consequences on their health.

The geographic differences in expenditures on different providers could arise because of the relative availability of different facilities in urban and rural areas. One can only pay for what is available. Hence, the urbanites spent more in public and private hospitals as well as pharmacy shops and laboratories, which are found more in the urban areas. Conversely, more money was spent on PMDs in the rural areas. However, the higher expenditures in urban areas could also be because the providers there charged higher fees than their rural counterparts bearing in mind that rural residents are usually poorer than the urbanites.

The fact that the major reason that people who needed healthcare services did not seek care was that either that the condition was not serious enough or they could not afford the cost of services is a pointer to the lack of financial risk protection in the health system in the study areas. Hence, the three main suggestions that the respondents gave for improving provision, utilisation and financing of healthcare services, which were provision of free services, subsidising healthcare and construction of more public hospitals should help in guiding design of programmes for enhancing financial risk protection of the health system in the study areas. A study showed that mortality rate falls from 1.1% to 6.9% for each 10% increase in public spending [5].

One limitation of the study is that the one-month recall period may not lead to very accurate collection of data on household health expenditures for ambulatory services and the longer recall period for in-patient stays is also subject to recall bias. Future studies should assess the real consequences for households of high levels of health expenditure. Such studies will require qualitative and observational design [34].

All in all the paper has provided additional knowledge on many issues such as information about level of healthcare visits and average expenditures on outpatient and inpatient care due to different diseases or health conditions. The findings also illustrated the differential use of different providers by different population groups. The pattern of expenditures by different SES quintiles and by people living in different geographic locations is instructive of the financing burden borne by different population groups. There was also evidence of differential patterns in provider choice by population group. The higher SES groups were associated with higher level of expenditures on private hospitals, public hospitals, pharmacy shops and laboratories. Conversely, decreasing SES was associated with more expenditure on PMDs. Expenditures on home treatment, PHC centres and herbalists were not associated with SES group, pointing at possibly more equitable payments and possibly service provision in the latter two types of providers, although the cell sizes were too small to detect differences. The differential expenditures paid by different quintiles could be as a result of providers charging more money to people that they knew or perceived to be well-off than they charged people that they knew or perceived to be poor [35].

In order to improve the provision and use of health services, people want increased free public health services, subsidising healthcare and construction of more public hospitals. People expressed a desire for increased free public health services, subsidised healthcare services, and the construction of more public hospitals. Reforms should identify constraints which impede the equitable distribution and access of free or subsidised public health services for the general population especially poor people and rural dwellers.

## Competing interests

The authors declare that they have no competing interests.

## Authors' contributions

OO conceived the study, participated in data collection and performed statistical analysis. BU, KH and CO participated in the design of the study and coordination. OO drafted the manuscript. All authors read and approved the final manuscript
